# Exercise-induced bronchoconstriction and atopy in Tunisian athletes

**DOI:** 10.1186/1471-2466-9-8

**Published:** 2009-02-05

**Authors:** Ridha Sallaoui, Karim Chamari, Abbas Mossa, Zouhair Tabka, Moktar Chtara, Youssef Feki, Mohamed Amri

**Affiliations:** 1 ISSEP-Sfax, Unité de Recherche "Les déterminants psychoculturels et biologiques de l'accès à la haute performance sportive", Sfax. Tunisia; 2 Research Unit ''Evaluation, Sport, Health" National Centre of Medicine and Science in Sport, CNMSS, El Menzah, Tunisia; 3 United Arabic Emirates National Olympic Commitee, Dubai, UAE; 4 Departement of Physiology and Lung Function Testing, Sousse Faculty of Medicine, University of Center, Sousse, Tunisia; 5 Laboratoire de Physiologie de la Nutrition, Faculté des Sciences de Tunis, El Manar 1060 Tunis, Tunisia

## Abstract

**Background:**

This study is a cross sectional analysis, aiming to evaluate if atopy is as a risk factor for exercise induced bronchoconstriction (EIB) among Tunisian athletes.

**Methods:**

Atopy was defined by a skin prick test result and EIB was defined as a decrease of at least 15% in forced expiratory volume in one second (FEV1) after 8-min running at 80–85% HRmaxTheo. The study population was composed of 326 athletes (age: 20.8 ± 2.7 yrs – mean ± SD; 138 women and 188 men) of whom 107 were elite athletes.

**Results:**

Atopy was found in 26.9% (88/326) of the athletes. Post exercise spirometry revealed the presence of EIB in 9.8% of the athletes including 13% of the elite athletes. Frequency of atopy in athletes with EIB was significantly higher than in athletes without EIB [62.5% vs 23.1%, respectively].

**Conclusion:**

This study showed that atopic Tunisian athletes presented a higher risk of developing exercise induced bronchoconstriction than non-atopic athletes.

## Background

Many athletes have breathing difficulties during or after athletic events and practice. Recently, several reports have delineated a significantly high prevalence of asthma symptoms in athletes [1-3]. Many triggers have been reported to induce the development of exercise-induced bronchoconstriction (EIB) also called exercise-induced asthma (EIA), notably the exposure to cold and dry air; humidity [4], thermal phenomena [5], and atopic status which also appears to be determinant. The reasons for this observation are still debated, but different mechanisms linked to the intensity of physical activity and atopy in athletes are probably involved [6,7]. Helenius et al. [7] indicated that atopic disposition increased the risk for asthma. Atopy is a form of immunological reactivity in which a reaginic antibody is readily produced in response to everyday exposure of a subject to common allergens in the environment [8]. Atopic reactivity can be verified by serological tests or by skin-prick tests to detect antigen-specific IgE antibodies [9].

Only a few studies have used objective methods to detect atopic allergy in athletes. In the study by Zwick et al.[10] 9 out of 14 swimmers had at least one positive reaction to the skin-prick test. Of 42 cross-country skiers studied by Larsson et al., [11], 29% were atopic according to the of results skin-prick test. Helenius et al. [7] found that 48% of the athletes and 36% of the control studied subjects were atopic according to the results of skin-prick tests. In North Africa, there are marked variations in the prevalence of asthma symptoms in the general population with up to 3-fold differences between the two periods pre- and post-1990; asthma prevalence showing a particularly marked increase during the last 10 years [12]. Recently, in the only report concerning the incidence of asthma in Tunisian athletes, Sallaoui et al. found that 13% of elite athletes (14/107) had EIA [13]. Tunisia as a North African country is characterized by a westernised lifestyle and epidemiological transition in which communicable diseases are receding and non-communicable diseases are emerging. We speculate that the westernized lifestyle and pollution are factors associated with the increased risk of atopy in Tunisians [12]. However, the prevalence of EIB associated with the increased risk of atopy among the Tunisian athletes is not known. Moreover, many athletes are reluctant to report asthma symptoms to a coach fearing that they will no longer be allowed to play or that they may be eliminated from the team or not be selected for events owing to their EIB. In that context, when discussing asthma with athletes, it is important for them to be reassured that with accurate diagnosis and proper management, they can still participate, even at the highest levels of competition.

The aim of this study was to establish risk of associations, specifically if atopy is as a risk factor for EIB among Tunisian athletes.

## Methods

### Study design

This study was conducted from November to mid-December 2003. During the testing days, the mean temperature and relative humidity were 10 ± 4°C (Range 6 – 14°C) and 45.6 ± 12% (Range 38 – 55%), respectively. Prior to exercising, a familiarisation session was conducted to collect demographic information and to familiarize the subjects to the study protocol and its field investigators. This session included a skin-prick test, a review of the study protocol, and a spirometry pre-test.

### Subjects

Three hundred and twenty-six athletes (188 males and 138 females, mean age 20.8 ± 2.7 years; range 17–24 years) of whom 107 were elite athletes training regularly (mean weekly training duration 18.3 ± 1.9 h, range 16 – 21 h), and 219 regional athletes regularly involved in regional championships (mean weekly training duration 10 ± 1.7 h, range 8 – 12 h) participated in the study. The athletes were divided into three groups according to their type of sport: (1) speed and power sport athletes (n = 114), endurance athletes (n = 54), and team sport athletes (n = 158). The main events of the speed and power athletes were weightlifting (n = 13), sprinting (100 to 400 meters, hurdles; n = 31), jumping (n = 15), taekwondo (n = 27), judo (n = 18), and gymnastics (n = 10). The endurance athletes were the long-distance runners competing in events from 800 meters to the marathon (n = 54). The team sport athletes were handball (n = 28), basketball (n = 25), soccer (n = 44), rugby (n = 33), and volleyball (n = 28) players. All subjects were clinically healthy and had no history of recent infection disease, asthma or cardiorespiratory disorders. Each participant underwent a skin-prick test and resting spirometry testing before and after exercise. The research protocol which was in accordance with the declaration of Helsinki, was approved by the Research Ethics Committee of the Faculty of Medicine, University of Sousse, Tunisia. All participants volunteered to participate to the study and were fully informed about the nature of the testing as well as the associated risks, and signed written consent forms before the experiments.

### Outcome measures

#### 1. Skin-Prick Test

Skin-prick test were performed with seven common air-borne allergens and positive (histamine dihydrochloride, 10 mg/ml) and negative (solvent) control solutions from ALK (Soluprick SQ, 10 histamine equivalent pricks (HEP); Allergologisk Laboratorium, Horsholm, Denmark). The allergens were as follows: birch, timothy, meadow fescue, and mugwort pollen and cow dander; the mite *Dermatophagoides pteronyssinus*; and spores of the mould *Cladosporium herbarum*. We considered an allergen-specific skin test response positive if the skin test panel was valid and the difference in the mean of the wheal's lengths and widths between the allergen-specific test and the negative control was at least 3 mm. A skin test panel was considered valid if the difference between the mean wheal diameters of the positive and negative controls was at least 1 mm.

#### 2. Measurement of forced expiratory volume in one second (FEV1)

FEV1 is the volume of air that can be forced out in one second after taking a deep breath; this value is considered as an important measure of pulmonary function that can indicate airway obstruction. The FEV1 test was carried out using a portable spirometer (Auto Spiro Pal; Minato Medical Science. Co., Ltd, Japan). The athletes performed the baseline test three times and the best result was recorded. The subject was seated comfortably, and she/he was instructed to take-in a full breath then to close the lips around the mouth piece and blow-out as hard and rapidly as possible. Inspiration had to be full and unhurried, and tested expiration had to be continuous without pause. The technique was demonstrated to each subject and the result was expressed in litres per second. FEV1 was measured at rest (pre-exercise) and at 0, 5, 10, 15, 20 and 30 min after completion of exercise. The subjects were diagnosed with asthma if any of the post-exercise FEV1 values was at least 15% lower than the pre-exercise FEV1 measurement. This level of 15% was chosen in accordance with the recommendations of the American Thoracic Society which suggested that this level is optimal for outdoor conditions [14,15].

#### 3. Exercise

The athlete ran for 8 minutes at 80–85% of the estimated maximum heart rate (HRmaxTheo) [16]. The exercise was performed without warm-up and the subjects ran in groups of four subjects along an outdoor track. During the run, the subjects were equipped with portable heart rate monitors (Polar S610, Oy, Kempele, Finland) set to record HR at 5 s intervals. Before exercise, each subject was informed about the range of HR at which she/he had to run according to her/his HRmaxTheo calculated according to Crapo et al.[15]: HRmaxTheo = (220-age) bpm. Target HR zones were pre-set on the programmable HR monitors so that the athletes were guided by audio alarms to keep their HRs between 80–85% of the estimated HRmax. Subjects attained the target zone in 45–60 s from the beginning of the run and the remainders of the 8 min run

### Statistical analyses

Student's t-test for independent samples was used to determine the differences between the averages of the variables in the two groups' variables (demographic variables and FEV1). A Chi-square test was used to assess the association between atopic and non-atopic athletes (percentage). Significance was set at an alpha level of 0.05, and all statistical analyses were conducted using the statistical package for the Social Sciences (SPSS, Version 13.0, SPSS Inc, Chicago, IL) and medical statistical methods (Tool of medical-statistical calculations allowing the evaluation of the value Diagnostic) [17].

## Results

All the 326 studied athletes completed the skin-prick test, the Spirometric test, and the running test. Anthropometric and lung function data recorded at rest are presented in Table 1. Exercise-induced bronchoconstriction was observed in 9.8% (32 out of 326) of the athletes and in 13% (14 out of 107) of the elite athletes. When individual data for each subject were pooled and analyzed by parametric statistical analysis (Student's t-test), no significant intergroup differences were observed in demographic variables or in pre-exercise FEV1 values (Table 1).

**Table 1:** Characteristics of the study population, Mean ± SD anthropometric and spirometric variables of the studied group

Variables	**S**	**EIB (+)**	**EIB (-)**
**Number**	**326**	32	294

Age (years)	20.8 ± 2 69	21.13 ± 2.64	20.56 ± 2.69

Weight (kg)	66.76 ± 9.72	66.51 ± 7.96	67.35 ± 8.26

Height (m)	1.75 ± 0.64	1.69 ± 0,14	1.76 ± 0.69

BMI (Kg/m^2^)	20 99 ± 3.66	20.89 ± 4.20	21.01 ± 3.59

FEV_1 _at rest (l/mn)	4.25 ± 0.86	4.03 ± 0.75	4.28 ± 0.88

Relative value of FEV1 (%)	121 ± 13	122 ± 18	119 ± 19

S, the study population; EIB (+) showed EIB (Exercise Induced Bronchoconstriction); EI (-) did not showed EIB; FEV1, forced expiratory volume in one second; Relative value of FEV1 = percentage of theoretical values [32]

**Figure 1:**
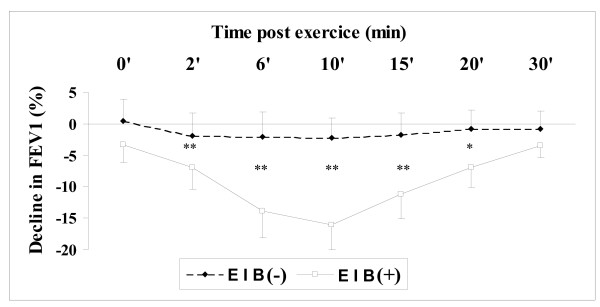
**Percentage change from baseline in forced expiratory volume in one second (FVE1) after exercise**. Spirometric results (mean) for the EIB(+) group (athletes who developed exercise-induced bronchoconstriciton, n = 32) and EIB(-) group (athletes with no observed EIB, n = 294). Intergroup comparisons of all data points indicated that EIB(+) had significantly lower FEV1 than EIB(-). *: p < 0.05; **: p < 0.001.

Figure 1 shows the Spirometric results (mean) for the EIB(+) group (athletes who developed exercise-induced bronchoconstriciton, n = 32) and EIB(-) group (athletes with no observed EIB, n = 294). Intergroup comparison indicated that athletes with EIB had significantly lower post-exercise FEV1 than the athletes without EIB (p < 0.001) with respective values of 16.85 ± 2.30% and 4.09 ± 3.51% of drop in FEV1.

Atopy according to skin-prick test results was found in 26.9% (88 out of 326) of all athletes, of these, 20 subjects presented EIB. Chi-square indicated that the frequency of atopy in athletes with EIB was significantly higher than in athletes without EIB [62.5% (20/32) vs 23.1% (68/294), respectively].

## Discussion

This study showed that atopy is a major risk factor for diagnosis of EIB in Tunisian athletes. Post-exercise spirometry revealed the presence of EIB in 9.8% (32/326) of the subjects, this prevalence of EIB is greater than in the general population [12]. Various studies in North Africa have been conducted to determine the prevalence of asthma but they have differed markedly, probably because of methodological considerations. The prevalence ranged from 2.4 to 3.4% in studies before 1990 and from 6 to 12% in the later ones [12]. In a study using "International Study of Asthma and Allergies in Childhood" (ISAAC) methodology conducted in the Tunis region on children aged 13–14 years, the prevalence was found to be 5.4% [12]. Only one recent study has been performed in Tunisian elite athletes showing EIB in 13% of the studied subjects [13]. The prevalence of asthma has ranged from 4% to 59% in various athletes' studies. This wide variation is mainly due to different types of training and training environments. Also differences in the definition and diagnosis of asthma may have had some impact on the mentioned range. An especially high prevalence of asthma has been found among those athletes competing in endurance events such as cycling, swimming, cross-country skiing, and long-distance running [2-4,6,7].

In order to estimate the prevalence of EIB in Tunisian athletes we chose to use a method for evaluating the respiratory condition of athletes, the most common is the change in FEV1 before and after exercise. This method is among the most widely used, and allows the study of a large number of individuals and avoids the task of recruiting subjects for laboratory testing. It also requires only a few minutes for testing and is relatively inexpensive. One of the limits of the present study is the difficulty to obtain standardized climatic conditions (temperature and humidity) but this is a common limitation of most outdoor studies.

It was found that 13% (14/107) of elite athletes suffered from EIB. Many studies have shown that the risk of developing EIB is increased in the elite athletic population [2-4,18,19]. The initial report about the incidence of EIA or EIB in elite athletes included the somewhat surprising finding that 11% of the US 1984 Summer Olympic Team experienced EIB [18]. In the 2000 summer Olympic Games in Sydney, 607 athletes (5.5% of the total) used inhaled B2-adrenoceptor agonists (β2-agonists) to treat active asthma [19]. This was a significant increase over the preceding 1996 summer Olympic Games in Atlanta, when only 383 athletes (3.6% of total) provided notification of use of B2-agonists [19]. Recently Alaranta et al. [2] reported that physician-diagnosed asthma and use of asthma medication were more common among Olympic level athletes than in the Finnish general population of the same age. Endurance athletes such as cross-country skiers, long-distance runners and swimmers suffered asthma more often than athletes in other events.

Among the objective methods for evaluating the respiratory condition of athletes, the most common is the change in FEV1 before and after exercise. Metacholine or histamine challenge tests are also used to determine the degree of EIB or airway hyper-responsiveness (AHR) in athletes. These standardized methods to evaluate the type and magnitude of airway response, i.e. 15 or 20% fall in FEV1 at specific provocation doses (PD15 or PD20) or provocation concentration (PC20) of methacholine or histamine were used to make the diagnosis of EIB. The prevalence of EIB obtained by these methods is much higher than that obtained in the present study, generally above the level of 20% (ranging from 19 to 76%) [20].

Helenius et al. [7] indicated that atopy is a major risk factor for EIB. In the present study, the diagnosis of atopy was based on the positive response to the skin tests underwent by all the subjects. A more objective evaluation by blood testing for the specific circulating IgE would probably allow the detection of more atopic subjects, but the skin-prick test is much more easier to administer ''on the field" in large populations. In total, 326 athletes underwent skin tests; atopy was identified in 26.9% (88/326). This prevalence of atopy among Tunisian athletes was not much different from the general population [21]. In the literature, some authors indicated that atopy in athletes may be partly related to exercising in extreme or particular environmental conditions which may favour its expression in the predisposed subjects [23,24].

An underestimation of the prevalence of atopy and EIB in our population might, however, come from the non-inclusion of swimmers. Indeed, it was recently shown that the swimmers are at higher risk for atopy and EIB development than other athletes [7]. In this context, in Europe and the USA, up to 1970, episodes of atmospheric pollution were frequently responsible for acute mortality epidemics by cardiovascular and respiratory diseases. The responsibility for such events was attributed to high concentrations of sulphur dioxide and particulate matter in the air of cities, usually due to unfavorable meteorological conditions and air stagnation. Urban air pollution is still highly prevalent in some developing countries [24]. Moreover, urban type pollution is still of major concern in Western countries with an increase in automobile-induced pollution. Throughout the world, indoor air pollution, tobacco smoking, and occupational exposures are of great concern [25].

Environmental factors involving the type and content of the inhaled air could play an important role. Even if most sports are practised in various air conditions all year long, many sports are predominantly practised either in cold, dry or humid air [26]. For athletes who train outdoors, the quality of the inhaled air varies and the presence of different pollutants may contribute to the development of EIB [26]. For the athletes who practise their sport in indoor areas, the exposure to such contaminants and to a variety of volatile organic compounds could contribute to certain respiratory problems [27]. Zwick et al. [10] indicated that swimming at high levels of performance constitutes the best example of chronic exposure to products and chloride derivatives of the chlorine used to disinfect swimming pools which may stimulate allergic mechanisms and facilitate the sensitisation to different allergens.

The present study has shown that that 62.5% of the atopic Tunisian athletes had EIB (20/32). Hence this prevalence is significantly greater than that in the athletes without EIB (62.5% vs. 23.1%) (p < 0.001). In a recent study, Helenius et al [7] demonstrated a close correlation between the onset of an EIB and the number of positive responses in the skin allergic test in athletes: for one to two positive allergic responses, the risk of an EIB is multiplied by 3.25 whereas for five positive reactions or more, this risk is multiplied by 4.69. Moreover, the severity of the EIB may moreover be correlated to the atopy score determined by adding the average diameters of the papules for all of the allergens evaluated [11,27]. In this regard Kaelin and Brandli [29] administered a questionnaire on allergy and exercise-related respiratory symptoms to 1530 Swiss athletes at national and international levels. Their study showed a significant correlation between atopy and respiratory symptoms. Helenius et al. [28] in another study including 58 runners belonging to the national Finnish team demonstrated that the occurrence of an EIB is strongly correlated with the seasonal variability. Whereas for certain athletes, the EIB happens only in winter, for others it occurs only in pollen-rich periods. These differences in bronchial responses may be explained by a shift of the pulmonary inflammatory state in the pollen-rich period. In this context, it was noted an increase of the number of eosinophils and the amount of eosinophil cationic protein in bronchiolo-alveolar lavage fluid in pollen allergic asthmatics during high pollen periods [30].

The best preventive measure of asthma occurrence is to achieve optimal control of the disease through athlete education, and environmental control. The education of athletes, their families and their coaches is an important component of the non-pharmacological management of EIB. To demystify this disease, athletes need to be informed that their condition is common among those in high-level competitions and will not limit their performance if it is treated adequately. Environmental control is important too, whenever possible. This applies to the home environment of the athletes, where avoidance of exposure to relevant allergens and to irritants should be suggested. It also applies to the training environment; for example, better ventilation systems in arenas and indoor pools could possibly help reduce the adverse effects of the numerous contaminants in suspension in ambient air.

Among the non-pharmacological approaches to EIB, a warm-up period prior to training or sports events can be effective in decreasing the degree of bronchoconstriction through induction of a refractory period, during which airways become less responsive to exercise [26]. A 10–15 minute warm-up at 60%VO_2max _can significantly reduce post-exercise asthma in athletes [31].

## Conclusion

This study suggests that an atopic field constitutes certainly a major risk factor for the development of an EIB in Tunisian athletes. The increasing prevalence of respiratory asthma-like symptoms in athlete is opening new paths for research into airway physiology in extreme conditions. We suggest that the athletes' medical staff should perform rigorous control (through a detailed checking of atopy symptoms and objective measurements such as resting spirometry) in order to allow early detection of eventual respiratory problems and provide adequate treatment in order to avoid EIB.

## Abbreviations

EIB: exercise-induced bronchoconstriction; EIA: exercise-induced asthma; FEV1: forced expiratory volume in one second; ISAAC: International Study of Asthma and Allergies in Childhood; AHR: airway hyper-responsiveness; PD: provocation doses

## Competing interests

Most of the authors or their research teams have received honoraria at various times for their involvement in advisory panels or meeting, and funding for research projects from the Ministere de l'enseignement supérieur, de la Recherche Scientifique et de la Technologie, Tunisia. However, the authors declare that they have no competing interests that affected their views expressed in this paper

## Authors' contributions

All authors were present for the full duration of the experiment and/of contributed by data analysis or discussion writing. All authors have had the opportunity to read and amend draft versions of the manuscript.

## Pre-publication history

The pre-publication history for this paper can be accessed here:


